# Plant-derived nutritional components in thyroid disease-related neuropsychiatric disorders: mechanistic insights and advances

**DOI:** 10.3389/fnut.2026.1695902

**Published:** 2026-02-19

**Authors:** Zihan Zhang, Meng-Xiang Xu, Nana Wang, Ruilin Shen, Jiayu Sun, Min Zha, Meizi Wang

**Affiliations:** 1 Nanjing University of Chinese Medicine, Nanjing, China; 2 Mengcheng County Hospital of Traditional Chinese Medicine, Bozhou Second Chinese Medicine Hospital, Bozhou, China; 3 Department of Endocrinology, Jiangsu Province Hospital of Chinese Medicine, Affiliated Hospital of Nanjing University of Chinese Medicine, Nanjing, China

**Keywords:** cognitive function, neuroinflammation, oxidativestress, plant-based nutrition, thyroid–brain axis

## Abstract

Plant-derived nutritional components represent a promising adjunctive strategy for addressing neuropsychiatric manifestations of thyroid disorders. Although conventional hormone replacement normalizes biochemical parameters, many patients continue to experience neurocognitive impairment and affective symptoms, underscoring the complexity of thyroid–brain axis regulation. This review synthesizes recent advances on how plant-based nutritional approaches may modulate oxidative stress, neuroinflammation, neurotransmitter homeostasis, and blood–brain barrier integrity, and evaluates their emerging clinical applicability and existing evidence gaps. Overall, plant-based interventions may complement standard therapy and improve neuropsychiatric outcomes, though larger prospective studies are needed to clarify their efficacy and identify suitable patient subgroups.

## 1 Introduction

Thyroid disorders are a unique intersection of endocrine and neurological health, and they are among the most widespread endocrine diseases worldwide (1, 2). Recent global estimates suggest that approximately 2.0–2.2 billion people worldwide have insufficient iodine intake, and iodine deficiency remains a leading preventable cause of thyroid dysfunction and related health problems (3). Within this global burden, hypothyroidism, hyperthyroidism, and autoimmune thyroid disease (AITD) affect hundreds of millions of individuals. The prevalence of hypothyroidism alone is estimated at 30% of the old population, while hypothyroidism is one of the most common thyroid diseases (4). The consequences of these conditions extend far beyond the endocrine system, with neurological and psychiatric effects emerging as critical yet under-recognized dimensions of the disease burden.

The effects of thyroid disorders on the central nervous system manifest across the entire lifespan: congenital hypothyroidism and iodine deficiency impair neurodevelopment in early life; overt and subclinical dysfunction in adults contribute to cognitive slowing and emotional instability; and in older individuals, hypothyroidism accelerates cognitive decline while hyperthyroidism increases anxiety and sleep disturbances (5–10). These findings indicate that thyroid dysfunction should be understood not only as an endocrine disorder but also as a neuropsychiatric condition. In recent years, the rising incidence of thyroid cancer further reinforces this perspective (11). Both thyroidectomy and radioactive iodine therapy frequently induce iatrogenic hypothyroidism, leading to persistent cognitive slowing, low mood, and fatigue, while the psychological burden of cancer increases the risk of anxiety and depressive symptoms (12, 13). Nutritional factors such as iodine and selenium have been investigated in relation to thyroid cancer risk, but epidemiological findings remain inconsistent, and the influence of cruciferous vegetables on thyroid function or thyroid cancer risk is similarly controversial, with current evidence still limited (14, 15). Thus, the potential role of nutritional factors in thyroid cancer development and post-treatment recovery remains a promising but as yet unconfirmed area.

The thyroid–brain axis represents a complex system of bidirectional communication between endocrine and neural networks. Thyroid hormones, particularly triiodothyronine (T3) and thyroxine (T4), are transported across the blood–brain barrier (BBB) by specialized carriers and are metabolically activated or inactivated by local deiodinase enzymes (16). Through genomic actions via nuclear thyroid hormone receptors—Thyroid Hormone Receptor Alpha (TRα) and Thyroid Hormone Receptor Beta (TRβ) and rapid non-genomic effects, thyroid hormones influence neuronal differentiation, synaptic plasticity, neurotransmitter turnover, and mitochondrial bioenergetics (17–21). This fine-tuned system ensures regional adaptation of thyroid hormone signaling to the metabolic and functional demands of different brain areas. When disrupted, the consequences extend to cognition, emotion, and behavior (22).

Current therapeutic paradigms focus primarily on pharmacological hormone replacement, most commonly levothyroxine (23). While highly effective at correcting serum TSH and T4, levothyroxine monotherapy does not always restore optimal neurocognitive performance. Approximately 10%−15% of patients on levothyroxine continue to report fatigue, mood instability, or memory complaints despite achieving biochemical euthyroidism (24–27). Likewise, treatment of hyperthyroidism with antithyroid drugs may control somatic symptoms but often leaves residual anxiety, irritability, or sleep disruption (28–31). This gap between laboratory normalization and patient wellbeing reflects the limitations of a pharmacocentric approach.

In response, attention has turned to complementary strategies that address the multifactorial nature of thyroid–brain dysfunction. Nutritional approaches, particularly those based on plant-derived foods, represent a promising avenue. Unlike single-agent pharmacological therapies, whole foods provide complex mixtures of micronutrients, antioxidants, polyphenols, and bioactive phytochemicals that act on multiple physiological targets (32–34). Selenium supports thyroid hormone metabolism and antioxidant protection; iodine provides the essential substrate for hormone synthesis; Mediterranean dietary patterns combine anti-inflammatory and neuroprotective foods; cruciferous vegetables supply phytochemicals such as sulforaphane and indole-3-carbinol; and adaptogenic herbs influence both stress regulation and thyroid activity (35–42). These interventions collectively offer not only mechanistic plausibility but also long-term feasibility and safety.

Nonetheless, caution is warranted. The evidence base for many of these interventions remains limited, uneven, or derived from non-thyroid populations (43). Selenium supplementation shows benefits in randomized controlled trials of Hashimoto's thyroiditis, but outcomes vary by baseline selenium status (44). Iodine demonstrates a *U*-shaped risk profile, with both deficiency and excess impairing thyroid and brain health (3, 45). Mediterranean diet adherence is associated with better cognition in population studies, but thyroid-specific trials are sparse (46). Adaptogens show promising effects in stress-related disorders, yet their role in thyroid disease is largely speculative (47). Recognizing these limitations is crucial to prevent overstated claims and to guide research toward areas of genuine promise.

**Figure 1:**
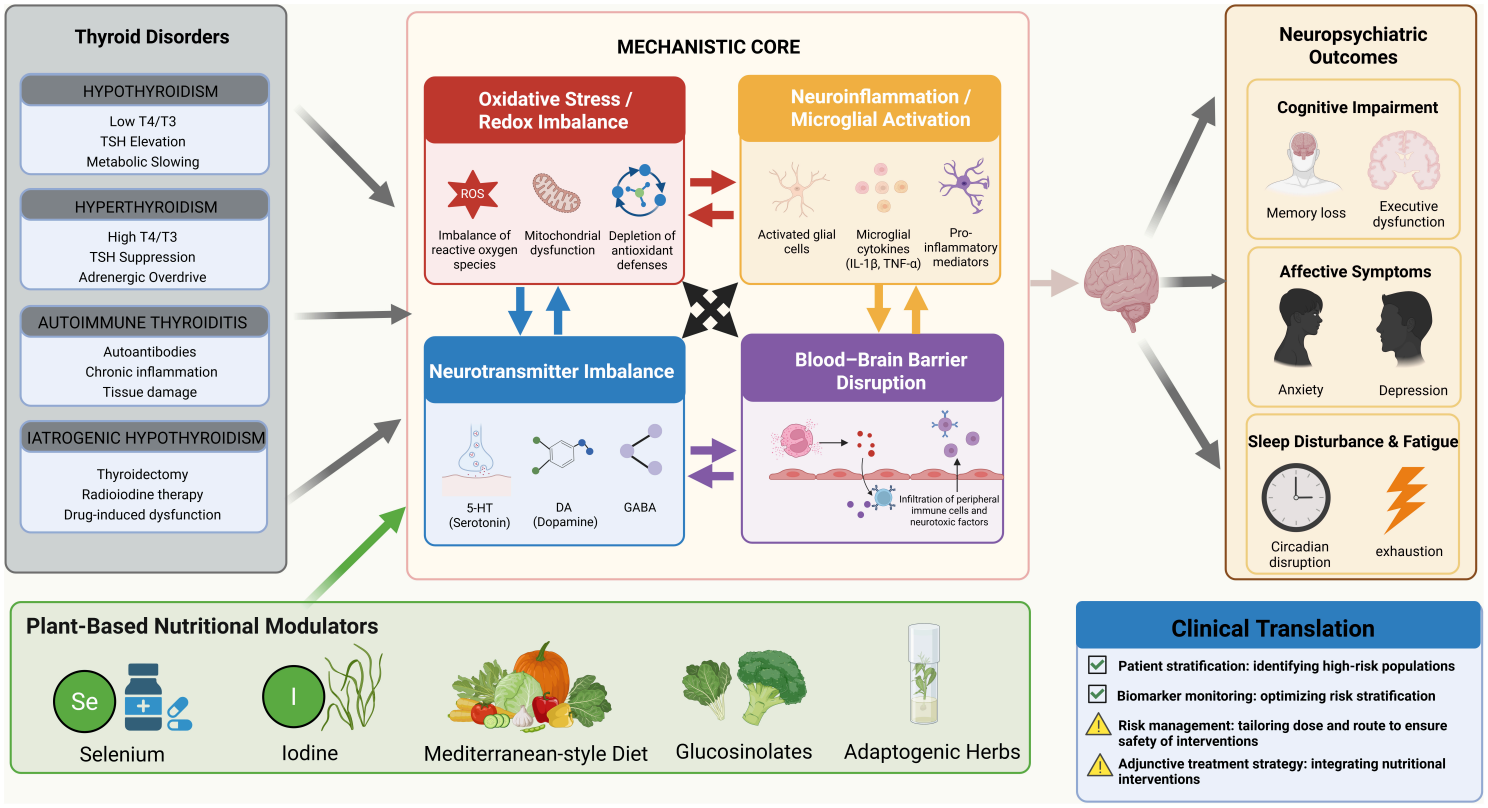
Integrated translational framework for thyroid–brain axis and plant-based nutritional modulation. TSH, thyroid-stimulating hormone; T3, triiodothyronine; T4, thyroxine; ROS, reactive oxygen species; IL-6, interleukin-6; TNF-α, tumor necrosis factor-α; 5-HT, serotonin (5-hydroxytryptamine); DA, dopamine; GABA, γ-aminobutyric acid. (Created with BioRender.com).

The purpose of this review is threefold: first, to describe the pathophysiological mechanisms by which thyroid dysfunction affects the brain; second, to examine the biological rationale for plant-based nutritional strategies targeting these mechanisms; and third, to critically appraise the clinical evidence supporting such interventions while acknowledging their risks and limitations. By situating plant-based nutrition within the broader landscape of thyroid–brain axis dysfunction, this review aims to provide a balanced framework for integrating nutritional strategies with conventional therapy, ultimately improving neurological outcomes and patient quality of life (Figure 1).

## 2 Literature search strategy and scope of evidence

This narrative review is based on a structured but non-systematic appraisal of the literature. Relevant studies were identified primarily through searches of PubMed, Web of Science, and Scopus, with a focus on publications from approximately 2000 to 2024. Priority was given to peer-reviewed experimental, translational, and clinical studies that directly addressed thyroid dysfunction–related neuropsychiatric outcomes, as well as plant-derived nutritional interventions with clear mechanistic relevance to oxidative stress, neuroinflammation, neurotransmission, or blood–brain barrier regulation.

Given the heterogeneity of evidence across this field, greater interpretive weight was placed on human studies, well-characterized animal models, and recent high-quality reviews. This approach was intended to enhance balance, contextual interpretation, and conceptual integration, rather than to provide a formal systematic synthesis.

## 3 Thyroid–brain axis pathophysiology and neurological manifestations

**Figure 2:**
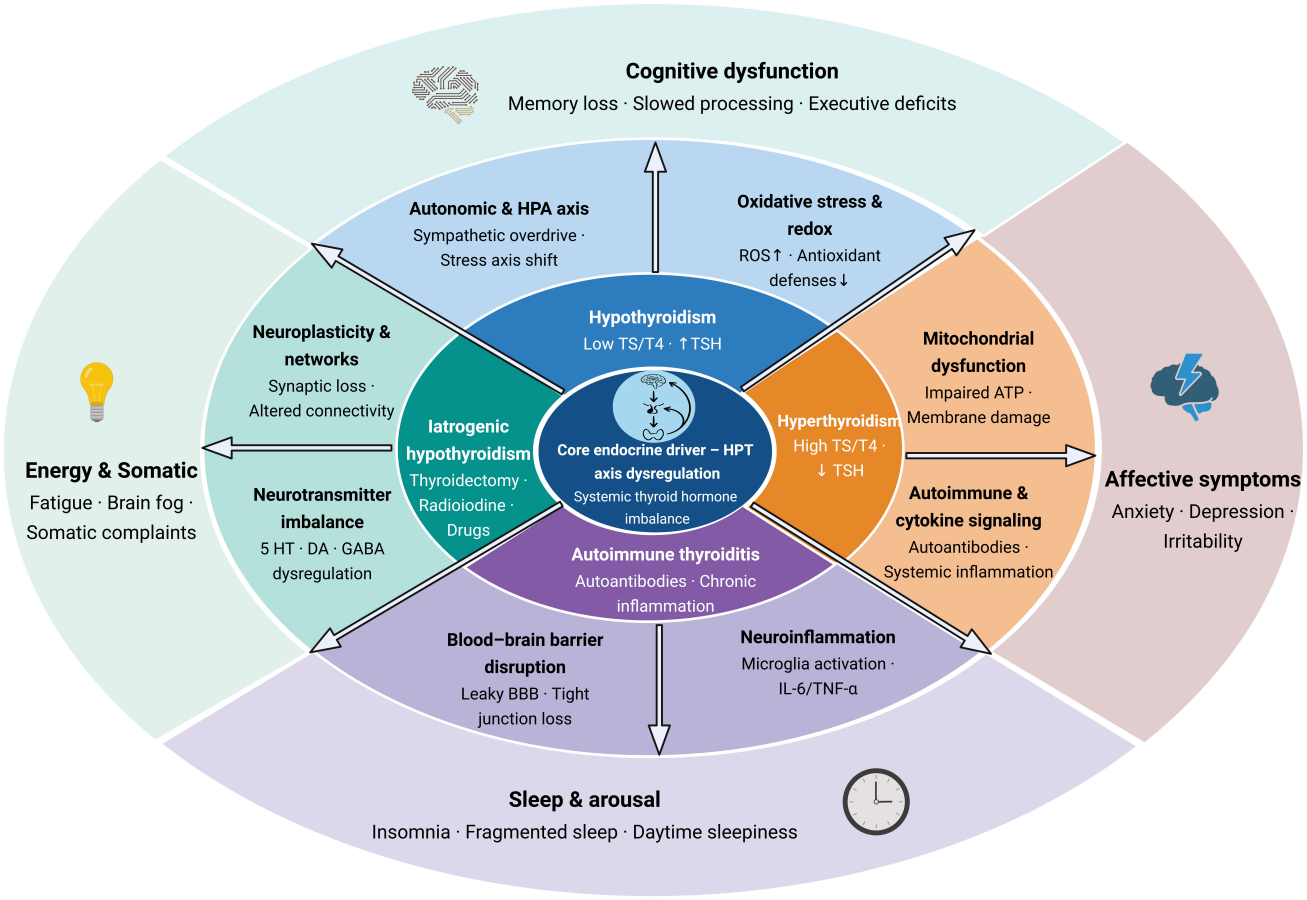
Multilayer thyroid-brain axis map: endocrine states, mechanistic pathways, and neuropsychiatric domains. HPT axis, hypothalamic–pituitary–thyroid axis; HPA axis, hypothalamic–pituitary–adrenal axis; TSH, thyroid-stimulating hormone; T3, triiodothyronine; T4, thyroxine; ROS, reactive oxygen species; ATP, adenosine triphosphate; 5-HT, serotonin (5-hydroxytryptamine); DA, dopamine; GABA, γ-aminobutyric acid; IL-6, interleukin-6; TNF-α, tumor necrosis factor-α; BBB, blood–brain barrier. (Created with BioRender.com).

The major mechanistic routes linking thyroid dysfunction to neuropsychiatric manifestations are summarized in Figure 2, highlighting disrupted hormone transport/activation in the brain, neurotransmitter imbalance, oxidative stress–neuroinflammation, and blood–brain barrier vulnerability. The neuropsychiatric manifestations of thyroid dysfunction and their associated mechanisms are summarized in Table 1.

**Table 1:** Neuropsychiatric manifestations of thyroid dysfunction.

**Disease type**	**Cognitive symptoms**	**Mood symptoms**	**Neurophysiological mechanisms**	**Neurochemical changes**	**Imaging features**	**Key references**
Hypothyroidism	Impaired working memory; slowed information processing; executive dysfunction; subjective cognitive slowing (“brain fog”)	Depression; apathy; anxiety; persistent fatigue	Reduced neuronal metabolic activity; impaired synaptic plasticity; abnormal myelination; reduced local activation of thyroid hormones in the brain	Decreased serotonin synthesis; reduced dopamine availability; impaired acetylcholine production	Reduced white matter integrity; decreased hippocampal volume; reduced cortical gray matter	(4, 7, 71–80, 83)
Hyperthyroidism	Impaired attention; memory disturbances; accelerated but inefficient cognitive processing; paradoxical cognitive impairment	Anxiety; irritability; emotional lability; panic symptoms; manic-like features	Neural hyperexcitability; dysregulated calcium signaling; enhanced sympathetic nervous system activity; disrupted sleep–wake architecture	Increased catecholamine signaling; enhanced calcium channel activity; reduced neuronal calcium buffering capacity	Increased cerebral blood flow; elevated cerebral glucose metabolism; reduced functional network efficiency	(7, 28–31, 84–93, 97–103)
Autoimmune thyroiditis	Mild to moderate cognitive impairment; reduced attention and processing speed; subjective memory complaints	Depression; anxiety; emotional instability; fatigue	Chronic neuroinflammation; microglial activation; immune-mediated neuronal stress; disruption of synaptic signaling	Increased pro-inflammatory cytokine activity; altered monoamine neurotransmission	Subtle structural brain changes; altered functional connectivity; region-specific cortical vulnerability	(7, 44, 109, 118–122, 206, 238)
Iatrogenic hypothyroidism	Reduced concentration; memory inefficiency; cognitive slowing following medical intervention	Depressive symptoms; emotional blunting; fatigue; reduced stress tolerance	Abrupt thyroid hormone deprivation; impaired neuroplastic adaptation; altered hypothalamic regulation	Reduced neurotransmitter synthesis secondary to thyroid hormone deficiency	Diffuse functional hypometabolism; reversible changes in cortical and subcortical activity	(12, 13, 23, 62, 79, 100)

### 3.1 Thyroid hormone neurobiology and brain function

Thyroid hormones are essential regulators of brain development and function. During fetal and early postnatal life, adequate maternal thyroid hormone supply is critical for neurogenesis, neuronal migration, synaptogenesis, and myelination (48). Even transient maternal hypothyroxinemia has been associated with lower IQ and impaired executive function in offspring (49). In adulthood, thyroid hormones continue to shape brain physiology, with effects on neurotransmission (50), energy metabolism (51), and synaptic plasticity (52).

The entry of thyroid hormones into the central nervous system depends on specialized transporters at the BBB (53). Monocarboxylate transporter 8 (MCT8) is the primary carrier for T3 and T4, while organic anion transporting polypeptide 1C1 (OATP1C1) facilitates T4 transport (18). Mutations in MCT8 cause Allan–Herndon–Dudley syndrome, a devastating neurodevelopmental disorder characterized by profound cognitive and motor impairment (19, 54). Even in acquired thyroid disease, regional differences in transporter expression may explain the selective vulnerability of hippocampal and cortical circuits to hormone fluctuations (55).

Within the brain, deiodinase enzymes modulate local hormone availability (56). Type 2 deiodinase (DIO2), expressed in astrocytes and tanycytes, converts T4 to active T3, ensuring a sufficient local hormone supply despite systemic variation (57, 58). Type 3 deiodinase (DIO3), expressed in neurons and glia, inactivates T3 and T4, generating reverse T3 and diiodothyronine (59–61). Together, these enzymes create microenvironmental gradients of thyroid hormone signaling, tailoring activity to the specific demands of each brain region. Importantly, systemic euthyroidism does not guarantee intracerebral euthyroidism; impaired DIO2 activity, for instance, has been linked to persistent cognitive symptoms in patients on levothyroxine therapy (62, 63).

At the cellular level, thyroid hormones act through nuclear receptors TRα and TRβ to regulate transcription of genes involved in neuronal metabolism, mitochondrial biogenesis, and cytoskeletal organization (64, 65). TRα is highly expressed in the hippocampus, cortex, and amygdala, regions critical for learning, memory, and emotion, while TRβ predominates in the cerebellum and striatum, contributing to motor coordination (66, 67). Non-genomic actions include rapid modulation of ion channels, neurotransmitter release, and calcium signaling, allowing real-time adjustments in excitability (68). These mechanisms collectively highlight the indispensable role of thyroid hormones in maintaining brain homeostasis.

### 3.2 Hypothyroidism and central nervous system dysfunction

Hypothyroidism affects the central nervous system in ways that extend beyond metabolic slowing. Patients commonly experience neurocognitive deficits such as reduced working memory, slower processing speed and impaired executive function, together with affective symptoms including depression, apathy and anxiety, which heighten the overall neuropsychiatric burden (7). Subclinical hypothyroidism, though milder, has been associated with reduced attention and an increased risk of affective symptoms, particularly in older adults (69, 70).

The neurochemical basis of these symptoms is multifactorial. Reduced thyroid hormone signaling downregulates tryptophan hydroxylase, impairing serotonin synthesis and contributing to depressive symptoms (71, 72). Dopaminergic pathways are similarly affected, with reduced tyrosine hydroxylase activity leading to diminished dopamine in the basal ganglia and prefrontal cortex, manifesting as anhedonia, reduced motivation, and psychomotor retardation (73, 74). Cholinergic dysfunction, mediated by decreased choline acetyltransferase activity, disrupts attention and short-term memory (75).

Neuroimaging studies corroborate these findings. Diffusion tensor imaging shows reduced fractional anisotropy in white matter tracts (76). Volumetric analyses demonstrate reductions in hippocampal and cortical gray matter, consistent with impaired synaptic plasticity (77, 78). Importantly, some of these structural abnormalities persist despite levothyroxine replacement, reflecting the inadequacy of systemic therapy in restoring intracerebral hormone balance (79, 80).

In pediatric populations, congenital hypothyroidism and untreated iodine deficiency remain major causes of intellectual disability worldwide (81). Even transient hypothyroidism during critical developmental windows can produce lasting deficits in IQ, attention, and language acquisition (82). In the elderly, hypothyroidism is associated with accelerated cognitive decline and an increased risk of Alzheimer's disease, raising the possibility that impaired thyroid signaling may act as a modifiable risk factor for dementia (83).

### 3.3 Hyperthyroidism and neurological hyperactivity syndromes

Hyperthyroidism, in contrast, produces a state of neural hyperexcitability (84). Patients often present with anxiety, irritability, tremors, restlessness, and sleep disturbances. Paradoxically, memory and concentration are frequently impaired despite an apparent acceleration of thought (85, 86). Affective manifestations include panic attacks, mood lability, and in some cases, manic-like episodes (87). These symptoms reflect adrenergic hypersensitivity and calcium dysregulation (88, 89). Excess T3 increases β-adrenergic receptor density in cortical and limbic regions, amplifying catecholamine responses and contributing to hyperarousal (90–92). This heightened adrenergic signaling disrupts prefrontal regulation of attention and limbic modulation of emotion (93).

Concurrently, T3 upregulates L-type calcium channels while reducing buffering proteins such as calbindin, creating a vulnerability to excitotoxicity (94–96). Hippocampal neurons are particularly affected, linking calcium dysregulation to paradoxical memory impairment (97, 98). Sleep architecture is also disrupted, with reduced Rapid Eye Movement (REM) sleep, increased awakenings, and poor sleep quality (99, 100). Functional imaging studies show increased cerebral blood flow and glucose utilization but decreased network efficiency, suggesting a mismatch between metabolic demand and functional connectivity (101–103). These abnormalities persist in some patients despite the normalization of thyroid hormone levels, again pointing to the limitations of conventional therapy in fully reversing neural instability.

### 3.4 Thyroid-specific oxidative stress and neuroinflammation

Oxidative stress and neuroinflammation are key mechanisms linking thyroid dysfunction to neuronal injury. In hypothyroidism, reduced thyroid hormone signaling first produces a redox imbalance marked by decreased synthesis of selenoproteins such as glutathione peroxidases and thioredoxin reductases (104–107). Lower glutathione peroxidase activity weakens the brain's ability to clear hydrogen peroxide and lipid peroxides, promoting reactive oxygen species accumulation and oxidative stress, which ultimately damages neurons (108, 109). These abnormalities persist even with a reduced metabolic rate, indicating that vulnerability arises mainly from impaired antioxidant defense rather than increased energy demand (110).

In hyperthyroidism, accelerated mitochondrial metabolism produces excessive reactive oxygen species (ROS), overwhelming endogenous antioxidant defenses (111, 112). This oxidative burden damages neuronal membranes and mitochondrial DNA, impairing energy supply and synaptic plasticity (113). Iodine imbalance further complicates this picture. Deficiency limits hormone synthesis, leading to developmental and cognitive impairment (3, 114). Excess iodine, conversely, enhances thyroid peroxidase–mediated hydrogen peroxide generation, promoting autoimmune thyroiditis and systemic oxidative stress (115–117). Elevated pro-inflammatory cytokines such as TNF-α, IL-1β, and IL-6 can cross the BBB, activating microglia and amplifying neuroinflammation (118). BBB integrity itself is compromised.

Hypothyroidism reduces the expression of tight junction proteins, increasing permeability and facilitating immune cell infiltration (119). Microglial activation perpetuates cycles of inflammation, oxidative injury, and neuronal loss, particularly in hippocampal and cortical regions (120–122). These cascades provide a mechanistic basis for persistent cognitive and mood symptoms that remain unresolved by endocrine therapy alone.

## 4 Plant-based nutritional strategies for thyroid–brain axis support

Given that oxidative stress, neuroinflammation, and neurotransmitter disturbances are central drivers of thyroid–brain axis dysfunction, dietary and plant-derived nutritional components that can modulate these interconnected pathways have a clear theoretical rationale. Many bioactive compounds exhibit antioxidant, anti-inflammatory, hormone-modulating, and barrier-stabilizing properties, providing a biologically plausible foundation for nutritional interventions (Figure 2). The mechanisms of key plant-derived nutrients involved in the thyroid–brain axis are summarized in Table 2.

**Table 2:** Mechanisms of key plant-derived nutrients in the thyroid–brain axis.

**Nutrient**	**Primary plant sources**	**Thyroid-level actions**	**Direct brain actions**	**Recommended intake**	**Safety considerations**	**Evidence quality**	**Key references**
Selenium	Brazil nuts; sunflower seeds; mushrooms	Cofactor for type 1, type 2, and type 3 iodothyronine deiodinases; reduction of thyroid peroxidase antibody levels; antioxidant protection in thyroid tissue	GPx synthesis promotion; thioredoxin reductase activation; neuronal oxidative stress protection; indirect regulation via hormones	200 μg/day (selenium-deficient areas)	Narrow therapeutic window; >400 μg/L; selenium toxicity risk; requires serum monitoring	Moderate quality (multiple randomized controlled trials)	(35, 44, 104–108, 195–198)
Iodine	Kelp; nori; seaweed varieties; iodized salt	Essential substrate for triiodothyronine and thyroxine synthesis; regulation of thyroid peroxidase enzymatic activity	Neurodevelopmental protection	150 μg/day (region-dependent)	*U*-shaped risk curve; excess induces autoimmunity; high variability	High quality (epidemiological studies)	(3, 36, 45, 140–145, 213–215)
Sulforaphane	Broccoli; kale; cabbage	Mild thyroid suppression at high doses	Nrf2 pathway activation; glutathione synthesis promotion; neuroinflammation inhibition	Safe from food sources, 30–40 mg/day	Safe with adequate iodine; requires proper cooking	Low–moderate quality (animal + *in vitro*)	(15, 170–176)
Indole-3-carbinol	Cruciferous vegetables	Regulation of estrogen metabolism; potential modulation of thyroid signaling	Anti-inflammatory signaling; mood stabilization support	Through food intake	Cooking reduces impact	Low quality (mechanistic studies)	(15, 177–179, 183)
Olive polyphenols	Extra virgin olive oil	Anti-inflammatory effects via nuclear factor kappa B pathway inhibition	Reduced neuroinflammation; blood–brain barrier protection; cognitive function support	20–30 ml/day extra virgin olive oil	Generally safe	Moderate quality (Mediterranean studies)	(157, 159, 160, 165, 166, 203–206)
Omega-3 fatty acids	Flaxseed; walnuts; chia seeds	Regulation of hypothalamic–pituitary–thyroid axis; anti-inflammatory actions	Membrane fluidity regulation; neurotransmitter balance; anti-depressant effects	Alpha-linolenic acid 1–2 g/day	Anticoagulant drug interactions	Moderate–high quality (depression studies)	(162, 165, 203–205, 208–212)

### 4.1 Selenium as a dual regulator of thyroid and brain function

Dietary selenium intake plays a pivotal role in sustaining optimal thyroid and neural function (123). Major sources include Brazil nuts, seafood, lean meats, eggs, and whole grains, with their selenium content largely dependent on soil concentration and regional variability (124, 125). Selenium is arguably critical micronutrient for maintaining thyroid–brain axis integrity (126). Incorporated into a unique class of selenoproteins, this trace element supports both endocrine regulation and neuronal defense (104). Within the thyroid, three iodothyronine deiodinases (DIO1, DIO2, and DIO3) depend on selenium for their enzymatic activity (127). DIO2, in particular, enables local T3 generation in astrocytes and tanycytes, ensuring brain regions maintain sufficient hormone activity even during systemic fluctuations (57, 128). Impaired selenium status disrupts this intracerebral conversion, potentially explaining why some patients continue to report “brain fog” despite normalized serum T4 (129).

In addition to hormone metabolism, selenium-dependent enzymes provide antioxidant protection (130). GPx (Glutathione peroxidases) neutralize hydrogen peroxide and lipid hydroperoxides, protecting neuronal membranes from peroxidative injury (131). Thioredoxin reductases maintain redox homeostasis and mitochondrial integrity (132). Selenoprotein P, the main selenium transport protein in plasma (133), delivers selenium to the brain via receptor-mediated transcytosis across the blood–brain barrier (134). The neural tissue exhibits a selective uptake of selenium, highlighting its crucial role in the evolutionary process of human cognitive resilience.

Mechanistically, selenium deficiency may exacerbate thyroid-related neurological dysfunction through three pathways: impaired deiodinase activity, weakened antioxidant defense, and disrupted selenium transport (134–136). Conversely, adequate selenium intake may stabilize both thyroid hormone activationand neuronal redox status (137, 138). Yet selenium's therapeutic range is narrow: deficiency undermines neuroendocrine function, while chronic excess can provoke selenosis with neurological symptoms of its own (139).

### 4.2 Iodine and brain-targeted hormone regulation

Iodine is indispensable for thyroid hormone synthesis and, through this role, exerts profound indirect effects on the brain (140). As the essential substrate for thyroglobulin iodination, iodine availability dictates systemic T4 and T3 production (141, 142). Deficiency leads to hypothyroxinemia and, in severe cases, congenital iodine deficiency syndrome characterized by irreversible cognitive impairment (143, 144). Even mild iodine insufficiency in adults may produce subtle deficits in memory and executive function, highlighting the sensitivity of brain tissue to thyroid hormone fluctuations (145).

At the same time, iodine excess introduces distinct risks. Thyroid peroxidase requires hydrogen peroxide to incorporate iodine into thyroglobulin (146–150). In states of high iodine intake, increased hydrogen peroxide generation enhances oxidative stress within thyroid follicles, promoting antigen presentation and autoimmune activation (151). These processes can indirectly harm the brain by destabilizing hormone supply and increasing systemic inflammation (151–153).

From a mechanistic perspective, iodine status creates a *U*-shaped risk curve for neurological outcomes: insufficient intake undermines hormone synthesis and cognitive capacity, while excess fosters autoimmunity and inflammatory cascades (145). Food-based iodine sources, particularly marine algae and sea vegetables, deliver iodine alongside polysaccharides and polyphenols that may buffer absorption and provide additional neuroprotective benefits (154, 155). However, their highly variable iodine content raises concerns about both under- and over-consumption (156).

### 4.3 Mediterranean dietary pattern as a multi-pathway intervention

The Mediterranean diet (MD) is not a single-nutrient strategy but a holistic dietary pattern that integrates multiple neuroprotective and thyroid-supportive components. Characterized by high intake of fruits, vegetables, legumes, nuts, seeds, and extra virgin olive oil, with moderate fish consumption and limited processed foods, the MD embodies a nutritional matrix well-suited to addressing thyroid–brain dysfunction (157, 158). Table 3 provides an overview of how core Mediterranean diet components support thyroid–brain axis function.

**Table 3:** Multi-pathway protective mechanisms of mediterranean diet components in thyroid–brain axis function.

**Dietary component**	**Specific foods**	**Thyroid benefits**	**Neuro- protective mechanisms**	**Synergistic effects**	**Implement- ation guidelines**	**Supporting evidence**	**Key references**
Extra virgin olive oil	Cold-pressed olive oil	Anti-inflammatory effects; endocrine regulation	Oleocanthal-mediated anti-inflammatory action; hydroxytyrosol-related neuroprotection	Fat-soluble vitamin carrier; polyphenol enhancement	20–30 ml daily; avoid high-heat cooking	PREDIMED trial (cognitive improvement)	(159, 160, 165, 204)
Fatty fish	Sardines; mackerel; salmon	Selenium and iodine supply; anti-inflammatory omega-3 fatty acids	Eicosapentaenoic acid and docosahexaenoic acid–mediated antidepressant effects; membrane fluidity regulation; neuroplasticity promotion	Omega-3 and polyphenol synergy; high-quality protein support	2–3 times per week; 150 g per serving	Multiple cohort studies (cardiovascular + cognitive)	(162, 165, 204, 205)
Nuts	Walnuts; almonds; hazelnuts	Rich selenium content; antioxidant support	Alpha-linolenic acid conversion to eicosapentaenoic acid; vitamin E–mediated neuroprotection; magnesium ion balance	Multi-element synergy; healthy fat combinations	30 g daily; mixed intake	Observational studies (cognitive decline delay)	(161, 205, 208)
Leafy greens	Spinach; arugula; lettuce	Folate supply; antioxidant support	Homocysteine metabolism; neurotransmitter synthesis support; nitrate-mediated vascular effects	Multi-vitamin synergy; fiber-prebiotic action	200–300 g daily; dark green priority	Nutritional epidemiology (cardiovascular)	(157, 206, 209)
Whole grains	Oats; brown rice; quinoa	B-vitamin support; blood glucose stability	Neurotransmitter precursor provision; stable energy supply; gut–brain axis regulation	Fiber–polyphenol synergy; comprehensive metabolic support	Replace refined grains; 150–200 g per meal	Moderate quality evidence (metabolic syndrome)	(210–212)
Legumes	Lentils; chickpeas; black beans	Plant protein; trace elements	Vascular protection; blood glucose stability; mood regulation support	Protein complementation; fiber synergy	3–4 times per week; combine with grains	Limited direct evidence	(157, 205, 212)

Several features are mechanistically relevant. Olive oil provides polyphenols such as oleocanthal and hydroxytyrosol, which suppress NF-κB activation and reduce pro-inflammatory cytokine release (159, 160). Nuts and legumes contribute selenium, folate, and plant proteins that support deiodinase activity and neurotransmitter synthesis (161). Fish provides omega-3 fatty acids (EPA and DHA), which enhance synaptic membrane fluidity, promote anti-inflammatory lipid mediators, and may modulate hypothalamic–pituitary–thyroid (HPT) signaling (162). Fruits and vegetables provide anthocyanins and carotenoids, some of which can cross the blood–brain barrier or act through their metabolites to improve cerebral perfusion and activate endogenous antioxidant systems (163, 164).

Through these overlapping mechanisms, the MD simultaneously targets oxidative stress, inflammation, hormone metabolism, and cerebrovascular function, which are among the key processes disrupted in thyroid disorders. Its integrative nature reduces reliance on any single nutrient, instead fostering synergistic interactions. While large-scale observational studies consistently link MD adherence to better cognitive outcomes, direct interventional evidence in thyroid-specific cohorts remains scarce (165, 166).

### 4.4 Cruciferous vegetables: risk–benefit considerations

Cruciferous vegetables, including broccoli, kale, and Brussels sprouts, occupy a controversial position in thyroid nutrition (15). On the one hand, they contain glucosinolates, which can be metabolized into goitrogenic compounds such as goitrin and thiocyanate (SCN^−^), which competitively inhibit iodide uptake by thyroid follicular cells (167, 168). In iodine-deficient states, excessive consumption of raw crucifers may exacerbate hypothyroidism (169).

On the other hand, crucifers are rich sources of bioactive phytochemicals with demonstrated neuroprotective activity (170). Sulforaphane, generated from glucoraphanin upon chopping or chewing, potently activates the Nrf2 transcriptional pathway, increasing glutathione synthesis and enhancing neuronal resilience against oxidative injury (171–176). Indole-3-carbinol influences estrogen metabolism and dampens inflammatory signaling, potentially stabilizing mood and cognition (177–179). Folic acid, vitamin K, and fiber further support neurotransmitter synthesis and gut–brain interactions (180–182). The risk–benefit profile of cruciferous vegetable consumption in thyroid disease is outlined in Table 4.

**Table 4:** Risk–benefit assessment of cruciferous vegetables in thyroid disease.

**Vegetable type**	**Goitrogenic compounds**	**Neuro- protective components**	**Risk scenarios**	**Benefit scenarios**	**Safe consumption strategies**	**Clinical recommendations**	**Key references**
Broccoli	Glucosinolates leading to thiocyanates	Sulforaphane; vitamin C; folate	Large amounts consumed raw may inhibit iodine uptake	Activation of nuclear factor erythroid 2–related factor 2 signaling; induction of antioxidant enzymes; inhibition of neuroinflammation	Steam for 5–7 min; ensure adequate iodine intake	Safe in iodine-sufficient populations; moderate intake in hypothyroidism	(15, 167, 168, 170–176, 183)
Kale	Isothiocyanates; glucosinolates	Carotenoids; vitamin K; antioxidants	Caution in hypothyroidism, especially with raw intake	Vascular protection; cognitive support	Cook before eating; combine with iodine-rich foods	Monitor thyroid function; individualize intake	(15, 167, 168, 170, 180, 181)
Cabbage	Goitrin (isothiocyanates)	Vitamin C; dietary fiber; plant sterols	Higher risk in iodine-deficient populations	Digestive health support; immune modulation	Fermentation (e.g., sauerkraut) reduces goitrogenicity; moderate intake	Avoid large raw amounts; especially in hypothyroidism	(15, 168, 169, 183)
Radish	Thiocyanates; isothiocyanates	Vitamin C; digestive enzymes; dietary fiber	Potential interaction with thyroid medications	Digestive promotion; immune regulation	Separate intake from medications; moderate consumption	Consult physician if on thyroid medications	(15, 167, 168, 183)

Cruciferous vegetables thus exemplify the principle that dietary risks and benefits depend on context. Adequate iodine intake and proper preparation (steaming, boiling, fermentation) mitigate goitrogenic potential while preserving neuroprotective phytochemicals (15). In populations with sufficient iodine status, crucifers may serve as valuable contributors to thyroid–brain nutrition (183). However, evidence directly linking crucifer intake to neurological outcomes in thyroid patients is sparse, and further clinical investigation is warranted (183).

### 4.5 Adaptogens and botanical adjuncts

Adaptogens are plant-derived compounds that enhance stress resilience and endocrine stability, representing emerging candidates for thyroid–brain support (184, 185). Ashwagandha (*Withania somnifera*) is the most extensively studied, with preclinical models showing increased T4 production, and reduced cortisol levels (186). Withanolides, its active constituents, modulate GABAergic and dopaminergic pathways, offering potential benefits for mood and cognition (187, 188).

*Rhodiola rosea* provides another example, with salidroside and rosavin enhancing mitochondrial ATP production, improving oxygen utilization, and stabilizing monoamine neurotransmitters under stress (189, 190). Holy basil, schisandra, and ginseng exhibit additional antioxidant and anti-inflammatory properties, though evidence remains preliminary and variable depending on preparation (191–193).

Adaptogens may exert therapeutic effects on thyroid-related neurofunctional disorders primarily by modulating the hypothalamic–pituitary–thyroid (HPT) axis and stress-responsive neural circuits. Through attenuation of chronic hypothalamic–pituitary–adrenal (HPA) axis overactivation, adaptogens such as *Withania somnifera* can normalize thyrotropin-releasing hormone (TRH) and thyroid-stimulating hormone (TSH) signaling, thereby improving peripheral thyroid hormone bioavailability (186). Concurrently, their antioxidant, anti-inflammatory, and neurotrophic actions help protect neuronal integrity from stress-induced damage, collectively supporting recovery of thyroid–brain axis function and neurocognitive performance (47). However, significant uncertainties remain regarding dosing, standardization, and safety in long-term use. At present, adaptogens should be viewed as experimental adjuncts with promising but unproven relevance to thyroid–brain health (184, 194).

To avoid redundancy in the subsequent sections, this chapter has summarized the key mechanisms related to selenium, iodine, the Mediterranean diet, and adaptogens. The following chapters will therefore focus on clinical evidence and practical applications rather than reiterating mechanistic details.

## 5 Clinical evidence and limitations

### 5.1 Selenium in thyroid populations

Selenium has been one of the most intensively studied micronutrients in thyroid disease, with multiple randomized controlled trials (RCTs) examining its effects in Hashimoto's thyroiditis and other autoimmune conditions. Supplementation at doses around 200 μg/day has consistently reduced thyroid autoantibody titers, and several studies have reported parallel improvements in fatigue, mood, and overall quality of life (195–197). For example, women with Hashimoto's thyroiditis receiving selenium reported better emotional regulation and cognitive clarity in addition to biochemical changes (198).

There is substantial heterogeneity across selenium intervention studies, and the differences in outcomes can largely be attributed to variations in population characteristics, baseline nutritional status, and study design. First, the effectiveness of selenium supplementation is highly dependent on baseline selenium levels: reductions in autoantibody titers and improvements in mood or quality of life are more frequently observed in selenium-deficient regions or among individuals with low serum selenium, whereas trials conducted in selenium-replete populations often report limited or no benefit (123, 199–201). Second, heterogeneity in study endpoints also contributes to inconsistent findings. Some trials focused on biochemical markers such as autoantibodies or TSH, while others assessed fatigue, mood, or subjective cognitive complaints, making direct comparisons difficult (198, 202). In addition, differences in supplement formulation, dosage, sample size, and intervention duration—typically ranging from 3–6 months—further amplify variability among studies (198, 199). Overall, current evidence suggests that selenium supplementation may hold greater potential in individuals with low selenium status, whereas its generalized use remains to be validated in larger and more rigorously designed trials.

### 5.2 Mediterranean diet and cognitive outcomes

Direct evidence of the Mediterranean diet (MD) in thyroid patients is limited, but insights from large-scale nutritional trials provide potential indirect support (166, 203). The PREDIMED trial, which enrolled thousands of older adults at cardiovascular risk, demonstrated that MD adherence may improve memory and executive function and potentially delayed progression to mild cognitive impairment (204). Cohort studies across Mediterranean regions generally report that MD patterns are associated with lower rates of depression and cognitive decline, conditions highly relevant to thyroid populations (205–207).

However, caution is needed when extrapolating these findings to thyroid-specific populations. Most existing evidence on the MD is derived from general elderly or cardiovascular high-risk cohorts rather than patients with thyroid disorders, which contributes to considerable variability across studies. Cognitive or affective benefits are more evident in individuals with higher inflammatory or metabolic burden, whereas studies involving low-risk participants or short follow-up durations often report minimal effects (165, 208). Moreover, the assessment of MD adherence is influenced by cultural, socioeconomic, and lifestyle factors, and definitions of “high adherence” vary considerably across studies (209, 210). The predominance of observational designs and the presence of residual confounding further increase heterogeneity. Nevertheless, the biological plausibility supported by the polyphenols, omega-3 fatty acids, selenium, and antioxidant components characteristic of the MD suggests that it may represent a feasible and relatively safe adjunctive approach for thyroid-related neurofunctional impairment (157, 203, 211, 212). Future research should prioritize rigorously designed prospective trials in thyroid-specific cohorts to validate these potential benefits.

### 5.3 Iodine balance and neurological function

The necessity of iodine for brain development is unequivocal: deficiency during pregnancy remains a leading cause of preventable intellectual disability worldwide, and universal salt iodization programs have dramatically improved population-level outcomes (3, 213). In adults, however, the relationship is more complex. Epidemiological studies reveal a *U*-shaped association between iodine intake and cognition, where both deficiency and excess correlate with poorer outcomes. Populations with moderate, stable intake typically perform better on memory and attention tasks than those at either extreme (45, 214, 215).

For thyroid patients, this balance is especially delicate. Individuals with iodine deficiency may benefit from dietary augmentation through seaweed or fortified foods, but excessive intake can precipitate autoimmune thyroiditis or interfere with levothyroxine absorption. Intervention studies with iodine-rich foods such as seaweed show mixed effects. Some suggest modest improvement in thyroid hormone markers, while others report increased autoantibody titers (216–218). These divergent outcomes highlight that iodine is essential yet potentially harmful when intake exceeds physiological requirements. In practice, its neurological impact depends heavily on baseline status and underlying thyroid pathology.

### 5.4 Phytochemicals and botanical compounds

Evidence for phytochemicals and botanicals is emerging but remains preliminary. Curcumin has been investigated in autoimmune and inflammatory disorders, with several trials demonstrating reduced cytokine levels and improved quality-of-life scores (219–221). While these findings are not thyroid-specific, the overlap with autoimmune thyroiditis suggests possible relevance (222). Green tea catechins, particularly Epigallocatechin-3-gallate (EGCG), have shown improvements in insulin sensitivity and cognitive performance in metabolic cohorts, mechanisms that could benefit thyroid patients given their shared metabolic vulnerabilities (223–226). Berry-derived anthocyanins have been linked to enhanced memory and neuroplasticity in older adults, outcomes highly pertinent to thyroid-related cognitive complaints (227–229).

Adaptogens, including Ashwagandha and Rhodiola, add another layer of interest. Ashwagandha has been shown in a human RCT to normalize thyroid indices (TSH↓, fT4/fT3↑) in subclinical hypothyroidism, with exploratory clinical observations and preclinical evidence suggesting potential benefits for central complaints such as fatigue, affective symptoms and neurocognitive function (194, 230, 231). However, these studies are generally limited by small samples, short follow-up, and variability in botanical preparations (232–234). Safety considerations also arise: Ashwagandha has occasionally precipitated thyrotoxicosis, and curcumin may interact with anticoagulants (41, 42, 235).

Overall, phytochemicals and botanicals offer mechanistic plausibility and early clinical signals but lack the robust evidence base seen with micronutrients like selenium. They remain intriguing but experimental options for thyroid-related neurological dysfunction. Within this context, these early signals should be regarded as exploratory rather than definitive, and their potential benefits require confirmation through larger, rigorously designed randomized controlled trials.

## 6 Practical implementation and clinical integration

The translation of plant-based nutritional strategies into routine thyroid care requires more than theoretical plausibility or isolated clinical trials. Effective implementation depends on systematic assessment, personalization, and careful integration with conventional pharmacotherapy. An individualized assessment framework is outlined in Table 5. Given the chronic nature of thyroid disease and the persistence of neurological symptoms despite biochemical control, these strategies should be viewed as adjunctive rather than alternative interventions (236–238). Building on the preceding mechanistic and clinical evidence, this section focuses on the practical implementation of plant-based nutritional strategies in clinical settings, including patient selection, monitoring procedures, and safety management, with an emphasis on how these interventions can be applied in practice.

**Table 5:** Individualized assessment framework for plant-based nutritional interventions in thyroid disease.

**Assessment dimension**	**Specific indicators**	**Evaluation methods**	**Risk stratification**	**Intervention strategies**	**Monitoring schedule**	**Adjustment principles**	**Key references**
Thyroid function status	Thyroid-stimulating hormone; free triiodothyronine; free thyroxine; thyroid peroxidase antibodies; thyroglobulin antibodies	Laboratory testing	Overt thyroid disease; subclinical thyroid dysfunction; normal thyroid function	Individualized nutritional plan; preventive nutritional intervention; maintenance nutrition	Reassessment every 1–3 months	Adjustment of nutrient intake based on thyroid hormone levels	(7, 23, 27)
Neurocognitive symptoms	Memory; attention; executive function; mood status	Montreal cognitive assessment; depression rating scales; fatigue assessment tools	Severe symptoms; mild-to-moderate symptoms; no significant symptoms	Intensive neuro-nutrition support; standard nutritional intervention; preventive nutrition	Evaluation every 3–6 months	Symptom improvement guides intensity adjustment	(4, 7, 241–244)
Baseline nutritional status	Selenium status; iodine status; vitamin D status; vitamin B12 status; folate status	Serum or urine testing	Deficient; marginally insufficient; adequate	Targeted supplementation; dietary adjustment; maintenance intake	Reassessment every 6 months	Baseline nutritional status determines supplementation strategy	(3, 35, 36, 123, 199–201)
Geographic and environmental factors	Soil selenium content; iodized salt coverage; drinking water iodine concentration	Regional survey data; environmental monitoring	Selenium-deficient regions; marginal regions; nutrient-sufficient regions	Intensive supplementation; moderate supplementation; priority use of food-based sources	Annual assessment	Environmental changes guide strategy adjustment	(3, 81, 213, 217, 239, 240)
Genetic susceptibility	Type 2 iodothyronine deiodinase gene polymorphisms; selenoprotein gene variants; thyroid-related gene variants	Genetic testing (optional)	High susceptibility; moderate susceptibility; low susceptibility	Precision nutrition; personalized dosing; standard protocol	Stable genetic background supports long-term strategy	Genotype-guided individualized adjustment	(63, 135, 270, 271)
Drug interactions	Levothyroxine; antithyroid medications; other chronic medications	Medication history review; pharmacist consultation	High interaction risk; moderate risk; low risk	Dose timing separation; dosage adjustment; close monitoring	Reassessment when medication regimens change	Medication priority with nutrition as auxiliary support	(23, 245–248, 250, 251)

### 6.1 Individualized assessment

A cornerstone of nutritional integration is recognizing inter-individual variability. Selenium status, for instance, differs dramatically by geography: populations in low-selenium regions may benefit from targeted intake, whereas those in replete environments risk toxicity with supplementation (123, 199). Similarly, iodine requirements vary with baseline exposure; coastal populations with seaweed-rich diets may require restriction, while inland communities remain at risk of deficiency (217, 239, 240). Clinical implementation thus begins with an assessment of dietary patterns, geographic context, and, when feasible, biochemical status such as plasma selenium or urinary iodine concentration (200, 239).

The analysis of the nervous system is equally critical for nutritional integration. Not all thyroid patients experience cognitive or mood disturbances to the same degree. Standardized assessments of memory, attention, mood, and sleep provide a baseline to evaluate nutritional interventions (241–244). Identifying patients with persistent brain-related symptoms despite hormone replacement helps target those most likely to benefit from adjunctive plant-based strategies.

### 6.2 Integration with pharmacotherapy

Nutritional strategies must be aligned with conventional thyroid treatment to avoid unintended interactions. Levothyroxine absorption, for example, can be reduced by high-fiber meals, calcium- and iron-rich foods, and some polyphenols (245–247). Clinical practice should emphasize temporal separation—typically administering levothyroxine on an empty stomach, with a 30–60 min interval before food intake, and several hours before calcium or iron supplements (248).

Iodine intake poses further challenges. Excessive consumption of iodine-rich seaweed can precipitate autoimmune flares (249). Adaptogenic herbs, such as Ashwagandha, may stimulate thyroid hormone synthesis, raising the possibility of overtreatment when combined with replacement therapy (250, 251). Such interactions highlight the necessity of close monitoring of thyroid function tests when introducing nutritional adjuncts.

### 6.3 Risk monitoring and safety considerations

Safety remains a critical aspect of implementation. While whole foods generally have favorable profiles, concentrated supplements or botanicals carry risks (252–254). Chronic high selenium intake can result in selenosis, with symptoms including irritability, fatigue, and even neurological impairment (255, 256). Seaweed consumption may deliver iodine far above recommended daily allowances, especially with kelp species (257). Botanical preparations vary in quality, and contamination or inconsistent standardization can compromise safety (258).

Practical implementation therefore requires structured monitoring. Regular thyroid function tests (TSH, free T4, free T3) remain essential. For patients receiving selenium supplementation, periodic serum selenium or GPx activity may be informative in research settings (259). Patient-reported outcomes (mood, fatigue, cognitive clarity, sleep quality) provide complementary data that often reflect the real-world impact of interventions more accurately than laboratory values alone.

### 6.4 Collaborative care models

Successful implementation of plant-based strategies also depends on integration into broader care frameworks. Endocrinologists, dietitians, and primary care physicians each play distinct roles: endocrinologists provide oversight of hormone replacement and laboratory monitoring; dietitians offer expertise in food-based interventions and meal planning; and primary care providers facilitate long-term follow-up and lifestyle management (260).

Patient education is equally vital. Many individuals with thyroid disease experiment with dietary modifications based on anecdote or internet sources, risking imbalance or excess. Clear communication about evidence-based benefits and potential risks can empower patients to make informed choices while avoiding harmful extremes.

### 6.5 Practical positioning

In practice, plant-based nutritional interventions should be considered as long-term adjuncts for patients with persistent neurological symptoms despite optimized hormone replacement. They are not substitutes for levothyroxine or antithyroid medications but may provide incremental benefits by addressing oxidative stress, inflammation, or neurotransmitter imbalances that pharmacotherapy alone does not resolve (261, 262). Implemented thoughtfully, with attention to individual variability, drug–nutrient interactions, and safety monitoring, these strategies can complement conventional care and improve patient-centered outcomes.

## 7 Future directions and research priorities

The current evidence base for plant-based nutritional interventions in thyroid-related neurological dysfunction is promising yet incomplete (263). To transform preliminary findings into robust clinical guidance, future research must prioritize methodological rigor, targeted populations, and mechanistic clarity. Building on the current implementation strategies, this section shifts toward future research directions, with a focus on evidence gaps, methodological needs, and the potential value of emerging technologies in advancing the nutrition–thyroid–brain axis.

### 7.1 Large-scale randomized controlled trials

Current clinical trials are predominantly constrained by insufficient sample sizes, single-center designs, and short intervention durations. These methodological limitations significantly undermine the reliability and generalizability of the findings. Although selenium supplementation demonstrates potential benefits in reducing autoantibody levels, the existing body of research—primarily limited to short-term observations of 3–6 months—fails to establish a definitive causal relationship between decreased antibody titers and sustained functional improvements in the neurological system (44, 264). Mediterranean diet interventions have demonstrated cognitive protection in general populations, yet thyroid-specific RCTs are lacking (158, 203, 265).

Future studies should therefore adopt multi-center, adequately powered designs with neurological endpoints as primary outcomes rather than secondary observations. Cognitive testing batteries, validated mood scales, and neuroimaging modalities such as functional MRI could provide objective evidence of brain-specific effects (266–269). Stratification by baseline nutritional status (e.g., selenium or iodine repletion) would clarify which populations benefit most. By prioritizing neurological outcomes in thyroid cohorts, such trials could establish definitive evidence for or against plant-based interventions.

### 7.2 Precision nutrition and individual variability

Nutritional interventions are unlikely to exert uniform effects across all thyroid patients. Genetic polymorphisms, metabolic phenotypes, and comorbidities shape individual responses. For example, Studies by Castagna et al. (63) demonstrated that the DIO2 Thr92Ala polymorphism substantially reduces type 2 deiodinase activity, impairs local T4-to-T3 conversion, and is associated with lower serum T3 levels in patients treated with levothyroxine. Gawandi et al. (270) further reported a notable frequency of this variant in patients undergoing T4 suppression therapy, accompanied by alterations in thyroid hormone metabolism. These primary data suggest that carriers of the Thr92Ala polymorphism may experience inadequate intracerebral T3 availability despite standard replacement therapy, contributing to persistent symptoms. Variants in selenoprotein P may similarly alter selenium utilization, while polymorphisms in catechol-O-methyltransferase (COMT) and other metabolic enzymes could influence responsiveness to polyphenols (271–273).

Integrating nutrigenomics and metabolomics into clinical research offers a pathway toward precision nutrition. By correlating genetic profiles, metabolite signatures, and microbiome composition with intervention outcomes, researchers can begin to tailor plant-based strategies to individual needs (274–276). Such an approach would move beyond population averages toward personalized adjunctive care, maximizing benefit while minimizing risk.

### 7.3 The gut–thyroid–brain axis

The gut microbiome is increasingly recognized as a mediator of both endocrine and neurological health (277, 278). Dysbiosis has been linked to altered thyroid hormone metabolism, increased intestinal permeability, and heightened systemic inflammation (279). Certain microbial taxa participate in deiodination reactions or influence enterohepatic recycling of thyroid hormones. Meanwhile, microbial metabolites such as short-chain fatty acids (SCFAs) exert significant effects on neuroinflammation and neurotransmission-related pathways. In a primary study using germ-free mice, Braniste et al. (280) demonstrated that SCFAs restore the expression of tight junction proteins such as occludin and claudin-5 at the BBB, thereby reducing inflammation-associated permeability and indirectly modulating neuroimmune signaling. These findings suggest that alterations in SCFA levels may influence affective and cognitive manifestations in thyroid dysfunction by affecting BBB integrity and neuroinflammatory responses (281, 282).

Dietary interventions, particularly high-fiber plant foods, cruciferous vegetables, and polyphenol-rich fruits, are potent modulators of the microbiome (283–285). Exploring how such foods influence thyroid hormone homeostasis and brain outcomes through microbial pathways represents an underdeveloped but promising field. Longitudinal studies combining dietary interventions with microbiome sequencing and metabolomic profiling could illuminate causal links. Probiotic or prebiotic formulations tailored to thyroid patients may eventually complement plant-based diets, extending therapeutic potential through microbiome modulation (286).

### 7.4 Long-term safety and sustainability

Safety and sustainability remain underexplored dimensions of plant-based interventions. Chronic selenium supplementation risks toxicity, with case reports of selenosis highlighting the narrow therapeutic margin (287). Iodine intake from seaweed is highly variable and may exceed tolerable upper limits in certain species (288). Adaptogens, while generally safe in short-term studies, lack long-term safety data, and inconsistent standardization complicates reproducibility.

Beyond safety, sustainability must also be considered. Recommendations that rely heavily on specific foods (e.g., Brazil nuts or kelp) may be impractical or environmentally burdensome (289). Research should therefore evaluate not only clinical outcomes but also feasibility, accessibility, and ecological impact. Pragmatic trials embedded within routine care could assess adherence, patient acceptability, and integration with diverse cultural dietary practices. By balancing efficacy with safety and sustainability, future research can generate recommendations that are both scientifically sound and practically implementable.

## 8 Conclusions

Thyroid disorders frequently present with persistent neurological symptoms that extend beyond endocrine imbalance. Although conventional pharmacotherapy effectively normalizes biochemical parameters, cognitive and emotional deficits often remain unresolved. Plant-based nutritional strategies—including selenium, iodine, Mediterranean dietary patterns, and selected phytochemicals—offer biologically plausible and generally safe adjunctive approaches by targeting oxidative stress, neuroinflammation, and neurotransmitter dysregulation.

However, the strength of evidence varies substantially across these interventions. Selenium and iodine are supported by a relatively robust body of thyroid-specific experimental and clinical studies, whereas evidence for dietary patterns and phytochemicals is more often derived from short-term trials, small cohorts, or non-thyroid populations, as well as secondary syntheses such as reviews and meta-analyses. In the context of shared mechanistic pathways relevant to thyroid–brain interactions, these data remain biologically informative, yet they also highlight a critical gap in thyroid-specific primary research.

At present, plant-based nutritional strategies should be regarded as supportive rather than substitutive to standard endocrine therapy. Careful individual assessment, attention to nutrient–drug interactions, and appropriate monitoring are essential to ensure safety. Future large-scale, long-term, thyroid-focused clinical trials with neurological endpoints are required to establish efficacy, clarify patient subgroups most likely to benefit, and translate mechanistic plausibility into evidence-based clinical practice.
